# Abemaciclib plus fulvestrant in treating hormone-receptor positive, HER2-negative advanced breast cancer—comparing real-world outcomes in England to the MONARCH-2 trial

**DOI:** 10.1038/s41416-026-03396-z

**Published:** 2026-03-30

**Authors:** Jack Anderson, Sarah Lawton, Katherine Thackray, Emma Kipps

**Affiliations:** 1 National Disease Registration Service, Data and Analytics, NHS England, London, UK; 2 Breast Unit, Department of Oncology, The Royal Marsden NHS Foundation Trust, London, UK

**Keywords:** Hormonal therapies, Breast cancer, Outcomes research, Breast cancer

## Abstract

**Background:**

Abemaciclib plus fulvestrant was approved in Europe following publication of the MONARCH-2 trial and recommended to enter the NICE Cancer Drugs Fund for HR+/HER2− advanced breast cancer. We aimed to assess MONARCH-2 generalisability to England clinical practice using real-world NHS trust data.

**Methods:**

We identified patients receiving abemaciclib plus fulvestrant from April to December 2019 in the NHS England Blueteq and Systemic Anti-Cancer Therapy data, with follow-up to March 2024. We calculated overall survival (OS) from treatment initiation until death, and treatment-free survival (TFS) and chemotherapy-free survival (CFS) from initiation until post-discontinuation treatment or death (restricting CFS to chemotherapy). We measured outcomes using Kaplan–Meier methodology and compared to MONARCH-2.

**Results:**

Median OS was 25.9 months [95% CI: 23.7, 28.4] (*N* = 876), compared to 46.7 months (*N* = 446) in MONARCH-2. Differences in gender, age and performance status did not explain OS differences. Median TFS was 11.6 months [95% CI: 10.3, 12.5] compared to a median PFS of 16.9 months in MONARCH-2. Median CFS was 15.3 months [95% CI: 13.8, 16.7], compared to 25.5 months in MONARCH-2.

**Discussion:**

MONARCH-2 trial data are not generalisable to this real-world cohort, which had notably shorter OS, TFS and CFS that could not be explained by differences in measured patient characteristics.

## Background

Breast cancer is the leading cause of cancer-associated death in women globally [1]. In England, more than 70% of patients with metastatic breast cancer are hormone receptor-positive, and treatment in this setting aims to delay progression and extend survival [2]. Cyclin-dependent kinase 4/6 inhibitors, when used alongside endocrine therapy (ET), have significantly extended progression-free survival (PFS) and overall survival (OS) compared to ET alone in advanced hormone receptor-positive, HER2-negative (HR+/HER2−) breast cancer [3]. Combination treatment is now considered standard care in first- and second-line settings.

Abemaciclib, an oral small-molecule inhibitor, selectively inhibits cyclin-dependent kinases 4 and 6, which are instrumental in regulating the cell cycle. This inhibition prevents retinoblastoma protein phosphorylation, leading to cell cycle arrest in the G1 phase, which slows tumour cell proliferation and makes abemaciclib particularly effective in treating HR+ breast cancer [4].

MONARCH-2, a Phase 3 clinical trial comparing abemaciclib plus fulvestrant to fulvestrant alone in advanced breast cancer treatment, prompted the National Institute for Health and Care Excellence (NICE) to review the clinical and cost effectiveness of abemaciclib plus fulvestrant in treating advanced HR+/HER2− breast cancer [5]. Abemaciclib plus fulvestrant was commissioned through the Cancer Drugs Fund (CDF) from April to December 2019 [6, 7]. During this time, MONARCH-2 formed the primary data source answering clinical uncertainties around OS and treatment duration, while a real-world evaluation using the Systemic Anti-Cancer Therapy (SACT) dataset and Blueteq data served as the secondary data source. Both analyses contributed to the NICE technology appraisal [8]. The SACT dataset is a population-based resource of SACT activity reported by NHS trusts in England, collated by the National Disease Registration Service [9], part of NHS England. Linking SACT to NHS England’s Blueteq High Cost Drug System provides patient-level data on real-world treatment patterns and outcomes in the NHS in England [10].

While Phase 3 trials competently assess treatment efficacy and safety under controlled conditions, cohorts are often highly selective and may not reflect clinical practice. In contrast, NHS real-world data reflects diverse patient demographics, treatment histories and comorbidities, offering insights into efficacy in routine clinical practice. This study evaluates the generalisability of MONARCH-2 to real-world treatment settings in the NHS in England by reevaluating the real-world cohort reported to NICE during the CDF period, extending the follow-up to present updated OS and calculating further measures, including treatment-free survival (TFS) and chemotherapy-free survival (CFS), to support comparisons to MONARCH-2 [11].

## Methods

### Data source

Data for this study are based on patient-level information collected in the SACT dataset as part of the routine care of cancer patients. All individuals were identified as receiving abemaciclib plus fulvestrant for the treatment of advanced HR+/HER2− breast cancer after ET in the NHS England Blueteq system [10]. All applications from 2 April 2019 to 15 December 2019 were considered in this analysis. Cases were linked to the SACT dataset using the NHS number.

### Patient cohort

CDF applications for abemaciclib plus fulvestrant identified in the Blueteq system were deduplicated by reviewing CDF application identifiers, approval dates and minimum SACT treatment dates; treatment dates were verified through bespoke patient-level follow-up with NHS trusts. Patients were excluded if they received abemaciclib plus fulvestrant via an Early Access to Medicines Scheme (EAMS), if the treating NHS trust confirmed the patient did not receive treatment, or if the patient’s treatment records could not be ascertained within the SACT dataset.

### Overall survival (OS)

OS was calculated using the Kaplan–Meier methodology, considering the interval from a patient’s earliest abemaciclib plus fulvestrant treatment record in SACT to a patient’s date of death or to the censor date. All patients were traced for their date of death using the NHS Personal Demographics Service on 25 March 2024, and this date was used as the censor date if patients were still alive [12]. The minimum potential follow-up for patients in the real-world cohort was 51.3 months.

### Subsequent treatments

The SACT dataset was interrogated to identify each patient’s final abemaciclib plus fulvestrant treatment, after which the first post-discontinuation treatment was identified. Treatments were categorised by clinical review into chemotherapy, targeted therapy, hormone therapy, or non-breast cancer treatment (allowing for combinations of these categories), and the distribution of post-discontinuation treatments was summarised. Definitions of each treatment category are listed in Table S1.

### Treatment-free survival (TFS)

TFS was calculated using the Kaplan–Meier methodology. The TFS interval was measured from a patient’s first abemaciclib plus fulvestrant treatment date in SACT to the date of their first post-discontinuation regimen, their date of death, or the censor date, whichever came first. If a patient did not receive a subsequent treatment and was still alive at the time of tracing for a date of death, they were censored on 25 March 2024. This study uses TFS as a real-world proxy for PFS.

### Chemotherapy-free survival (CFS)

CFS was calculated using the Kaplan–Meier methodology. The CFS interval was measured using the same method as the TFS interval; however, only chemotherapy treatment was considered when identifying a patient’s first post-discontinuation regimen, as opposed to TFS, which included any post-discontinuation breast cancer SACT treatment. If a patient did not receive a subsequent chemotherapy treatment and was still alive at the time of tracing for a date of death, they were censored on 25 March 2024.

For both TFS and CFS analysis, patients who received a non-breast cancer treatment following abemaciclib plus fulvestrant (*N* = 2) were censored at the start date of the non-breast cancer treatment. Only patients who went on to receive a breast cancer treatment were recorded as having next treatment events in these analyses.

A breakdown of event definitions for OS, TFS and CFS is provided in Fig. S1.

### Comparing clinical trial and real-world outcomes

To establish differences in OS, TFS and CFS amongst patients treated in the NHS compared to those treated in the clinical trial, median estimates and 95% confidence interval estimates from the real-world cohort were descriptively compared to the median results from MONARCH-2.

### Subgroup analyses

To investigate differences between subgroups within the real-world cohort, OS analyses were also stratified by gender, broad age group (<65 and 65+), Eastern Cooperative Oncology Group performance status (PS) (PS 0–1, PS 2+, PS Unknown), and a Blueteq data item detailing previous ET. TFS and CFS were also stratified by previous ET.

## Results

### CDF cohort of interest

**Fig. 1:**
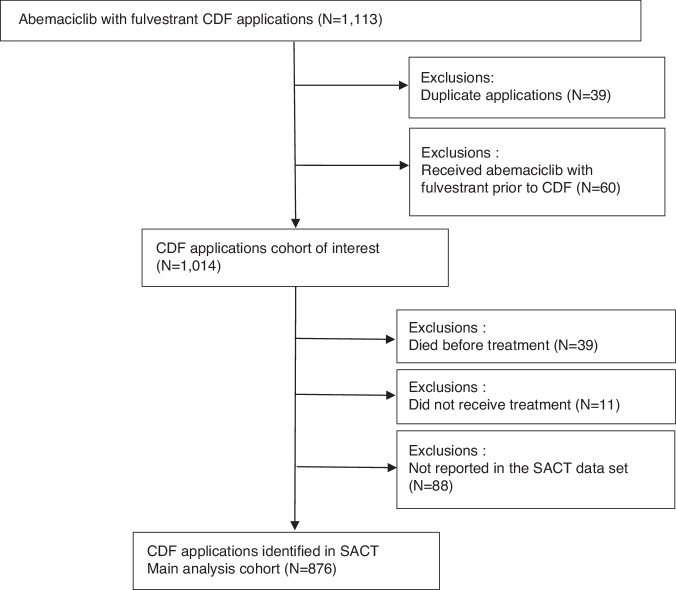
Cohort of interest. Derivation of the cohort of interest from all Cancer Drugs Fund (Blueteq) applications made for abemaciclib plus fulvestrant for the treatment of advanced HR+/HER2− breast cancer between 2 April 2019 and 15 December 2019.

Between 2 April 2019 and 15 December 2019, there were 1113 applications for CDF funding for abemaciclib plus fulvestrant for advanced hormone receptor-positive, HER2-negative breast cancer in the Blueteq database (Fig. 1). Following de-duplication, this related to 1074 unique patients. Sixty patients were excluded from these analyses due to having received abemaciclib plus fulvestrant prior to 2 April 2019 via an EAMS. Eleven patients did not receive abemaciclib, three of whom went on to receive chemotherapy in place of abemaciclib. Thirty-nine patients died before treatment, and 88 patients were missing from SACT. As a result, 91% (*N* = 876) of the expected cohort were identified and included in the real-world cohort for this analysis.

### Overall survival (OS)

**Fig. 2:**
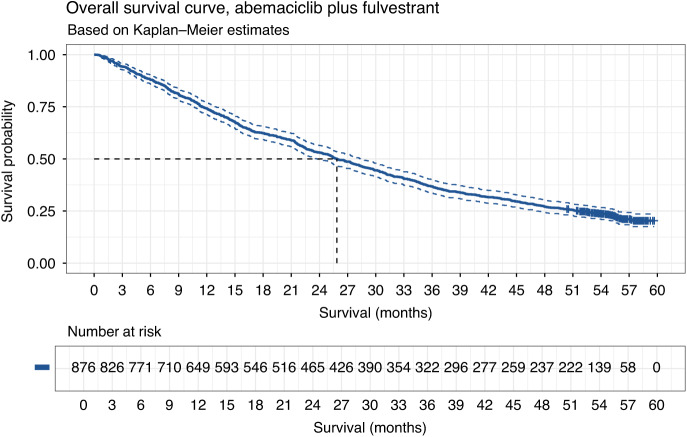
Overall survival. Overall survival (with 95% CIs) amongst the real-world cohort that received abemaciclib plus fulvestrant for the treatment of advanced HR+/HER2− breast cancer (*N* = 876).

Of the 876 patients with a treatment record in SACT, the maximum follow-up was 59.7 months. Figure 2 provides the Kaplan–Meier curve for OS, censored on 25 March 2024. The median OS was 25.9 months [95% CI: 23.7, 28.4]. OS was 74% [95% CI: 71%, 77%] at 12 months, 53% [95% CI: 50%, 56%] at 24 months, and 37% [95% CI: 34%, 40%] at 36 months (Table S2). The real-world cohort’s median OS was 20.9 months shorter than that of the MONARCH-2 cohort, which had a median OS of 46.7 months (*N* = 446) [11].

### Treatment-free survival (TFS)

A total of 522 patients (60%) who received abemaciclib plus fulvestrant were prescribed a further line of systemic therapy, compared to 76% of the MONARCH-2 cohort. Of these 522 patients, 64% received chemotherapy (*N* = 334) as their next line of treatment compared to 45% of patients in the MONARCH-2 trial. 23% (*N* = 121) of patients in the real-world cohort went on to receive a combination of targeted therapy and hormone therapy, and 11% (*N* = 59) of patients received hormone therapy. 1% (*N* = 4) of patients went on to receive a combination of chemotherapy and targeted therapy, less than 1% (*N* = 2) received targeted therapy only, and less than 1% (*N* = 2) of patients went on to be prescribed a non-breast treatment as the first post-discontinuation therapy (Table S1).

**Fig. 3:**
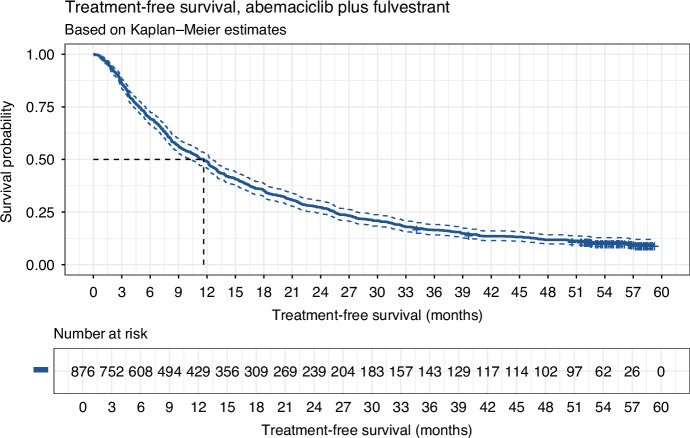
Treatment-free survival. Treatment-free survival (with 95% CIs) amongst the real-world cohort that received abemaciclib plus fulvestrant for the treatment of advanced HR+/HER2− breast cancer (*N* = 876).

The median TFS was 11.6 months [95% CI: 10.3, 12.5] (Fig. 3), while the median PFS in MONARCH-2 was 16.9 months [11]. TFS was 69% [95% CI: 66%, 73%] at 6 months, and 49% [95% CI: 46%, 52%] at 12 months (Table S2). The 3-year TFS rate was 16% [95% CI: 14%, 19%], notably lower than the 3-year PFS rate of MONARCH-2 (30%).

### Chemotherapy-free survival (CFS)

**Fig. 4:**
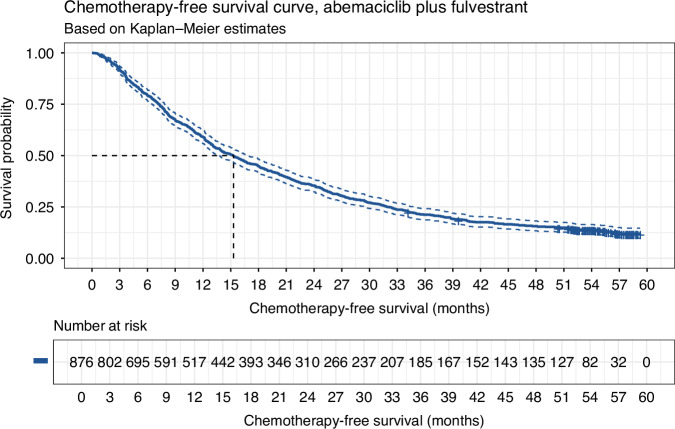
Chemotherapy-free survival. Chemotherapy-free survival (with 95% CIs) amongst the real-world cohort that received abemaciclib plus fulvestrant for the treatment of advanced HR+/HER2− breast cancer (*N* = 876).

51% (*N* = 444) of the real-world cohort were prescribed a post-discontinuation chemotherapy regimen. The median CFS for the real-world cohort was 15.3 months [95% CI: 13.8, 16.7] (Fig. 4), with a CFS rate of 59% [95% CI: 56%, 62%] at 12 months and 45% [95% CI: 42%, 48%] at 18 months (Table S2). Median CFS for the MONARCH-2 cohort was 25.5 months, 10.2 months longer than that of the real-world cohort.

### Subgroup analysis—gender, age and performance status

99% (*N* = 865) of the real-world cohort were female, and 1% (*N* = 11) were male (Table 1). All patients in MONARCH-2 were female. Subgroup analysis indicated that for the real-world cohort, there was no statistically significant difference in median OS between genders (Fig. S2, *p* value = 0.3).

**Table 1:** Patient characteristics of the real-world cohort that received abemaciclib plus fulvestrant for the treatment of advanced HR+/HER2− breast cancer (*N* = 876).

Patient characteristics^a^			
		*N*	%
Gender	Female	865	99%
	Male	11	1%
Age	<40	21	2%
	40–44	37	4%
	45–49	55	6%
	50–54	82	9%
	55–59	126	14%
	60–64	116	13%
	65–69	119	14%
	70–74	134	15%
	75–79	114	13%
	80+	72	8%
Performance status	0	273	31%
	1	416	47%
	2	66	8%
	3	4	<1%
	4	1	<1%
	Missing	116	13%
Previous endocrine therapy^b^	Has progressive disease whilst still receiving adjuvant or neoadjuvant endocrine therapy for early breast cancer, with no subsequent endocrine therapy received following disease progression	302	34%
	Has progressive disease within 12 months or less of completing adjuvant endocrine therapy for early breast cancer, with no subsequent endocrine therapy received following disease progression	32	4%
	Has progressive disease on 1st line endocrine therapy for advanced/metastatic breast cancer, with no subsequent endocrine therapy received following disease progression	542	62%

^a^Figures may not sum to 100% due to rounding.

^b^Data sourced from the NHS England Blueteq system.

The median age of the real-world cohort was 65 years, compared to 59 years in MONARCH-2. Subgroup analysis showed that in the real-world cohort, there was no statistically significant difference in OS between patients under the age of 65 and patients aged 65 and over (Fig. S2, *p* value = 0.54).

79% (*N* = 689) of patients had a PS of 0 or 1, 8% (*N* = 71) of patients had a PS of 2 or higher, and PS was missing for 13% (*N* = 116) of patients. MONARCH-2 was restricted to patients with a PS of 0 or 1. Subgroup analysis in the real-world cohort saw that the OS, TFS and CFS for patients with a PS of 2 or higher was significantly reduced compared to those for patients with a PS of 0 or 1 (Fig. S3, *p* value < 0.05). However, even when restricted to PS 0–1, the median OS of the real-world cohort was 28.5 months [95% CI: 26.2, 31.1], the median TFS was 12.2 months [95% CI: 11.1, 13.2] and the median CFS was 16.4 months [95% CI: 14.9, 18.6] which are all still shorter than those of MONARCH-2.

### Subgroup analysis—previous endocrine therapy

**Fig. 5:**
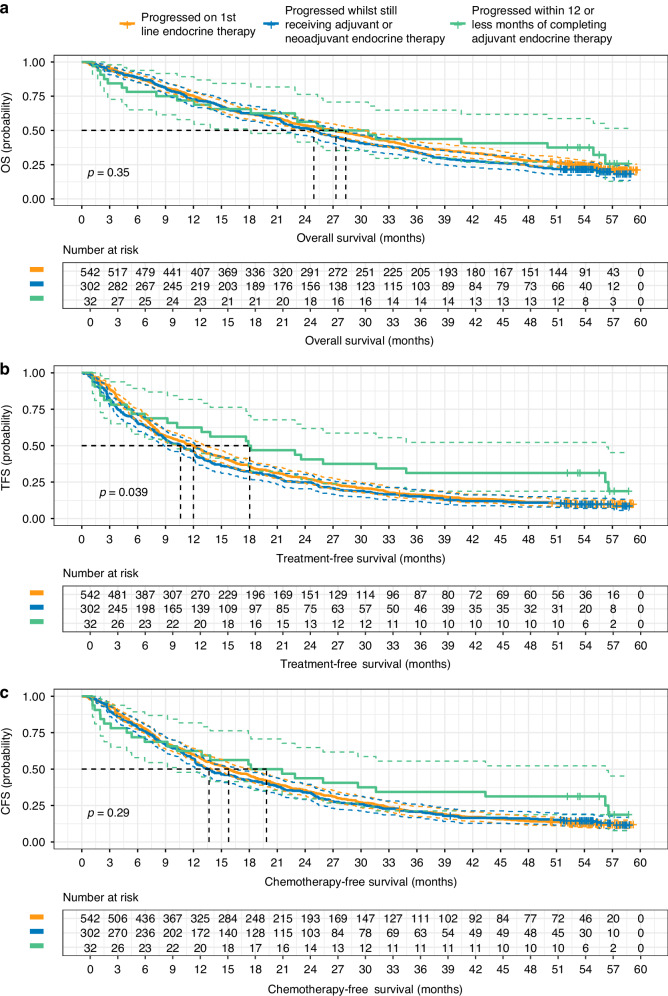
Survival outcomes by Blueteq previous endocrine therapy. Outcomes (with 95% CIs) stratified by Blueteq data item previous endocrine therapy amongst the real-world cohort that received abemaciclib plus fulvestrant for the treatment of advanced HR+/HER2− breast cancer, including **a** overall survival, **b** treatment-free survival and **c** chemotherapy-free survival (*N* = 876).

The distribution of previous ET in Table 1 shows that 34% (*N* = 302) of patients had progressive disease whilst receiving adjuvant or neoadjuvant therapy, 4% (*N* = 32) of patients had progressive disease within 12 or fewer months of completing adjuvant therapy, and 62% (*N* = 542) of patients had progressive disease on 1st line ET. In MONARCH-2, 25% of patients (*N* = 112) in the treatment arm had primary endocrine resistance, and 73% (*N* = 326) had secondary endocrine resistance [11]. In the real-world setting, the cohort of patients identified as having had progressive disease whilst receiving adjuvant or neoadjuvant therapy had the poorest outcomes across all key endpoints: median OS 24.9 months [95% CI: 21.7, 28.0]; median TFS 10.6 months [95% CI: 8.8, 12.2]; median CFS 13.7 months [95% CI: 12.2, 16.7] (Fig. 5).

## Discussion

This study compared the real-world outcomes of abemaciclib plus fulvestrant treatment in hormone receptor-positive, HER2-negative breast cancer patients in England with the treated cohort within the MONARCH-2 trial. The results highlight key differences between the clinical trial data and real-world evidence across OS, TFS and CFS, suggesting that the MONARCH-2 trial data are not generalisable to the real-world setting in the NHS in England. The median OS in the real-world cohort of patients treated within the NHS was 20.9 months shorter than the median OS reported in the MONARCH-2 trial. The real-world cohort was made up of older patients than MONARCH-2 and featured patients with ECOG PS 2+, while MONARCH-2 was generally younger and restricted to patients with ECOG PS 0–1 [11]. However, subgroup analyses conducted suggested this disparity cannot be attributed to differences in age or PS between the two cohorts.

The patient eligibility criteria were mostly concordant between the CDF and the MONARCH-2 trial with regard to the requirement that patients had a disease that progressed while on neoadjuvant or adjuvant ET, up to 12 months after adjuvant ET, or while receiving ET for metastatic disease. However, patients who had received any prior chemotherapy for metastatic disease were excluded from MONARCH-2 but were not excluded from the CDF eligibility criteria [5, 7]. Therefore, the real-world cohort may have been a more heavily pre-treated population, typically associated with a poorer OS [13], which may account for the differences in outcome seen between the two cohorts. Although the comorbidities of patients treated within the CDF are unknown, there are no exclusion criteria related to past medical history; this differs from MONARCH-2, in which patients with serious pre-existing conditions were excluded at the investigator’s discretion. Therefore, differences in comorbidities between the two groups may also have contributed to the observed differences in OS [14]. The potential for the real-world cohort to be more heavily pre-treated and have a higher prevalence of comorbidities is corroborated by a lower proportion of patients receiving post-discontinuation therapy in the real-world setting (60%) compared to in the MONARCH-2 trial (76%).

Similarly to OS, TFS and CFS were substantially shorter in the real-world population compared to the median PFS of MONARCH-2. In addition, of the patients who went on to receive post-discontinuation therapy, a higher proportion went on to receive chemotherapy in the real-world cohort (64%) than in MONARCH-2 (45%). Therefore, as well as being more likely to receive post-discontinuation chemotherapy, the real-world cohort was treated with chemotherapy earlier than in MONARCH-2, as the median CFS for the real-world cohort was 10.2 months shorter than in MONARCH-2. Notably, although both TFS and CFS were reduced in the real-world cohort compared to MONARCH-2, whether patients in the real-world cohort went on to receive subsequent treatment due to disease progression or due to an intolerance of abemaciclib plus fulvestrant is not captured in the data.

The more frequent and earlier transition to chemotherapy in the real-world cohort may reflect a greater prevalence of endocrine resistance, noting that the population in MONARCH-2 with primary resistance derived comparable, if not greater, benefit from treatment. 34% of patients treated in the NHS had progressive disease whilst receiving adjuvant or neoadjuvant therapy, a population in which there is likely to be a high proportion of primary resistance [15]. The proportion of patients with primary resistance to ET in MONARCH-2 was 25%, which may explain the slower transition to subsequent treatment following abemaciclib plus fulvestrant in the trial cohort. This is because while primary resistance may reflect intrinsic tumour characteristics, secondary resistance often develops due to adaptive changes following treatment [16, 17]. The distinction between primary and secondary resistance therefore guides the tailoring of subsequent therapies in clinical practice and may be a causative factor in the earlier transition to subsequent therapy seen in the real-world cohort [18, 19].

### Study limitations

While this study offers crucial insights, it is important to recognise its limitations. The first being the descriptive comparisons drawn between the real-world cohort and MONARCH-2, as this study’s source of comparator results for MONARCH-2 has only reported median endpoint values. Secondly, the real-world cohort was subject to missing data, particularly regarding ECOG PS and prior treatment history. Further, as the SACT dataset is focused on treatment activity, some patient characteristics on which MONARCH-2 is restricted could not be measured in the real-world cohort using this dataset alone. For example, while patients with some serious pre-existing medical conditions were not eligible for the MONARCH-2 trial, this study did not examine the burden of patient comorbidities in the real-world cohort. Additionally, this study used TFS as a proxy for PFS. It should be recognised that, in the real-world cohort, starting or delaying subsequent treatment is influenced not only by disease progression but also by factors such as toxicity, patient preference and local access policies. As such, while this measure does not exclusively reflect disease progression, it serves as the best approximation possible using the SACT dataset.

## Conclusion

This study compared real-world outcomes for abemaciclib plus fulvestrant in hormone receptor-positive, HER2-negative breast cancer patients treated within the NHS in England to those in the MONARCH-2 trial, highlighting notable differences in OS, TFS and CFS. These differences could not be explained by differences in age, gender, or PS between the real-world cohort and the MONARCH-2 trial. This variation underscores the need to consider real-world factors, such as patient comorbidities and treatment history, when assessing the generalisability of clinical trial data, and informs more effective treatment approaches for diverse patient populations in routine practice.

## Supplementary information

Supplemental Information

## Data Availability

SACT data are made available to properly authorised analysts and researchers under data access arrangements through the Data Access and Release Service (DARS). Available online at: http://digital.nhs.uk/services/data-access-request-service-dars#national-disease-registration-service-ndrs.

## Code availability

The code supporting this study’s results is not currently publicly available. Please contact the corresponding author to discuss code availability.
