# Supplementary material for: Abemaciclib plus fulvestrant in treating hormone-receptor positive, HER2-negative advanced breast cancer—comparing real-world outcomes in England to the MONARCH-2 trial
Source: Br J Cancer. 2026 Mar 30;134(10):1440–6. doi: 10.1038/s41416-026-03396-z (PMC13133171; doi:10.1038/s41416-026-03396-z)
Supplement: Supplementary file 1 — Supplemental Information [file 41416_2026_3396_MOESM1_ESM.docx]

**Supplemental Information**

This file includes supplemental information describing patient pathways following treatment with abemaciclib plus fulvestrant; overall survival amongst the real-world cohort stratified by patient characteristics; post-discontinuation therapy groupings; and overall survival, treatment-free survival, and chemotherapy-free survival rates across a 48-month follow-up period.


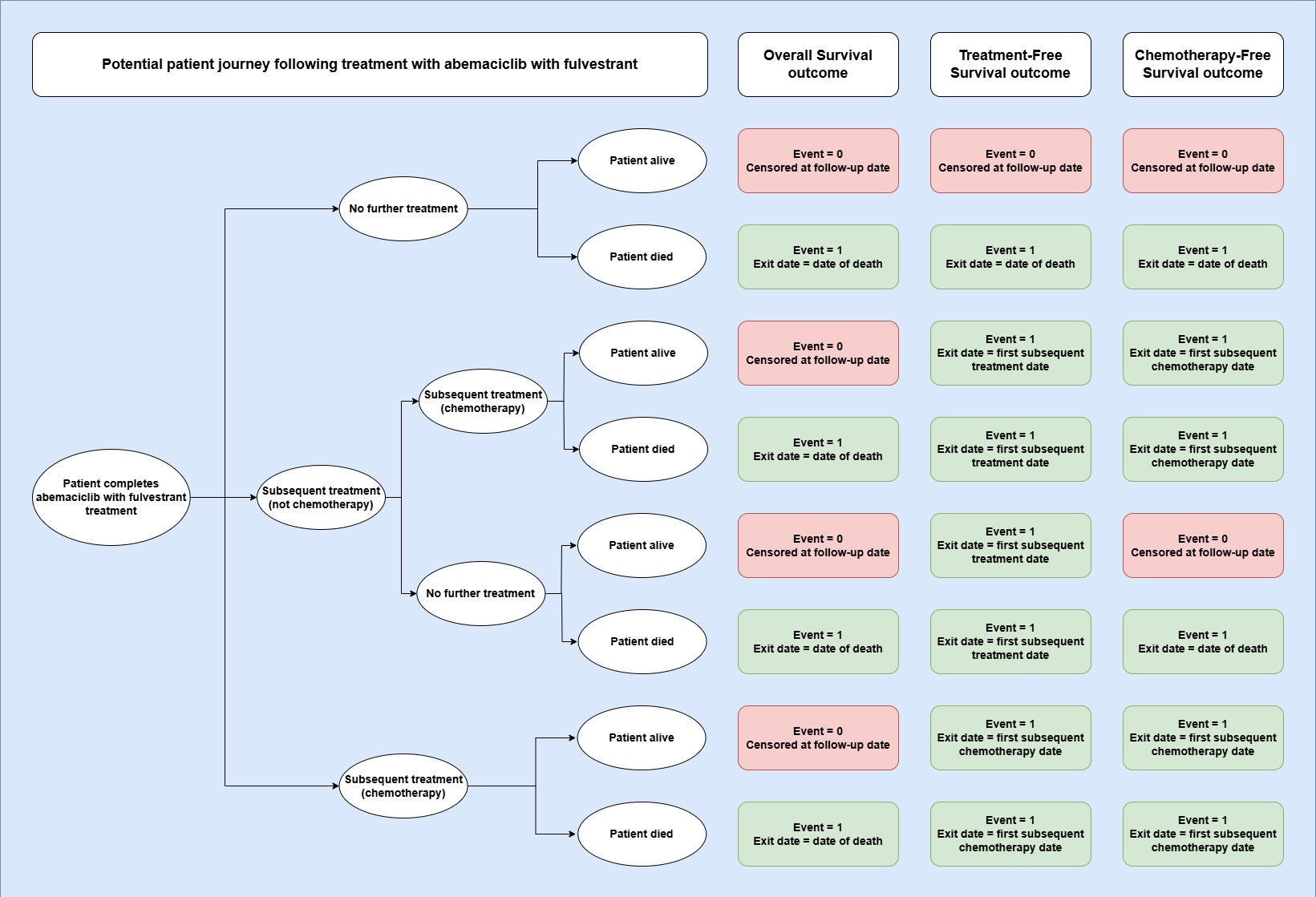
Figure S1 – Potential patient journeys following treatment with abemaciclib plus fulvestrant, and the associated event assignments for overall survival, treatment-free survival, and chemotherapy-free survival outcomes


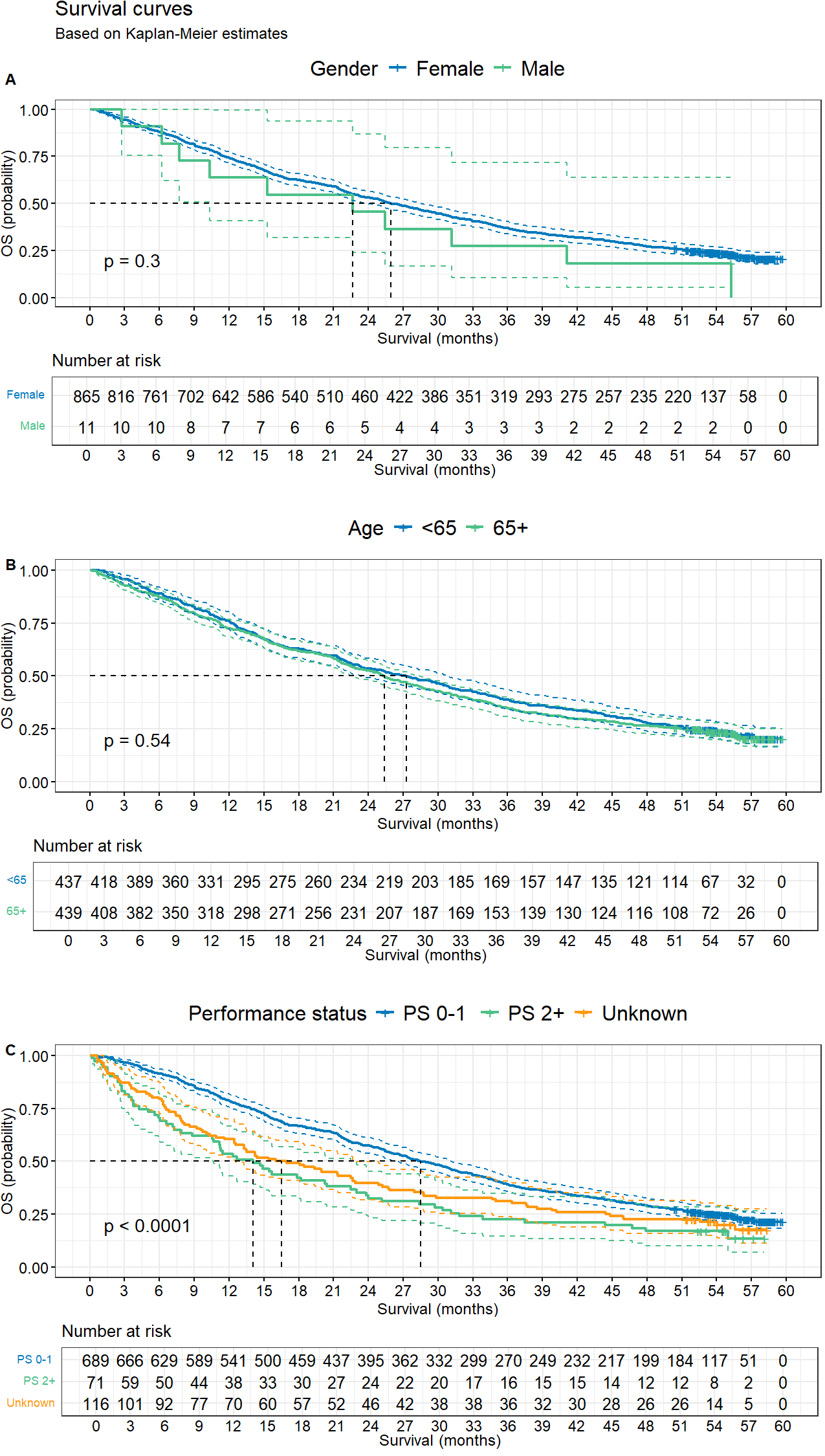
Figure S2 – Overall survival amongst the real-world cohort that received abemaciclib plus fulvestrant for the treatment of advanced HR+/HER2- breast cancer - stratified by (A) gender, and (B) age group (N = 876)

Figure S3 – Outcomes (with 95% CIs) stratified by Eastern Cooperative Oncology Group performance status amongst the real-world cohort that received abemaciclib plus fulvestrant for the treatment of advanced HR+/HER2- breast cancer, including (A) overall survival, (B) treatment-free survival, and (C) chemotherapy-free survival (N=876)


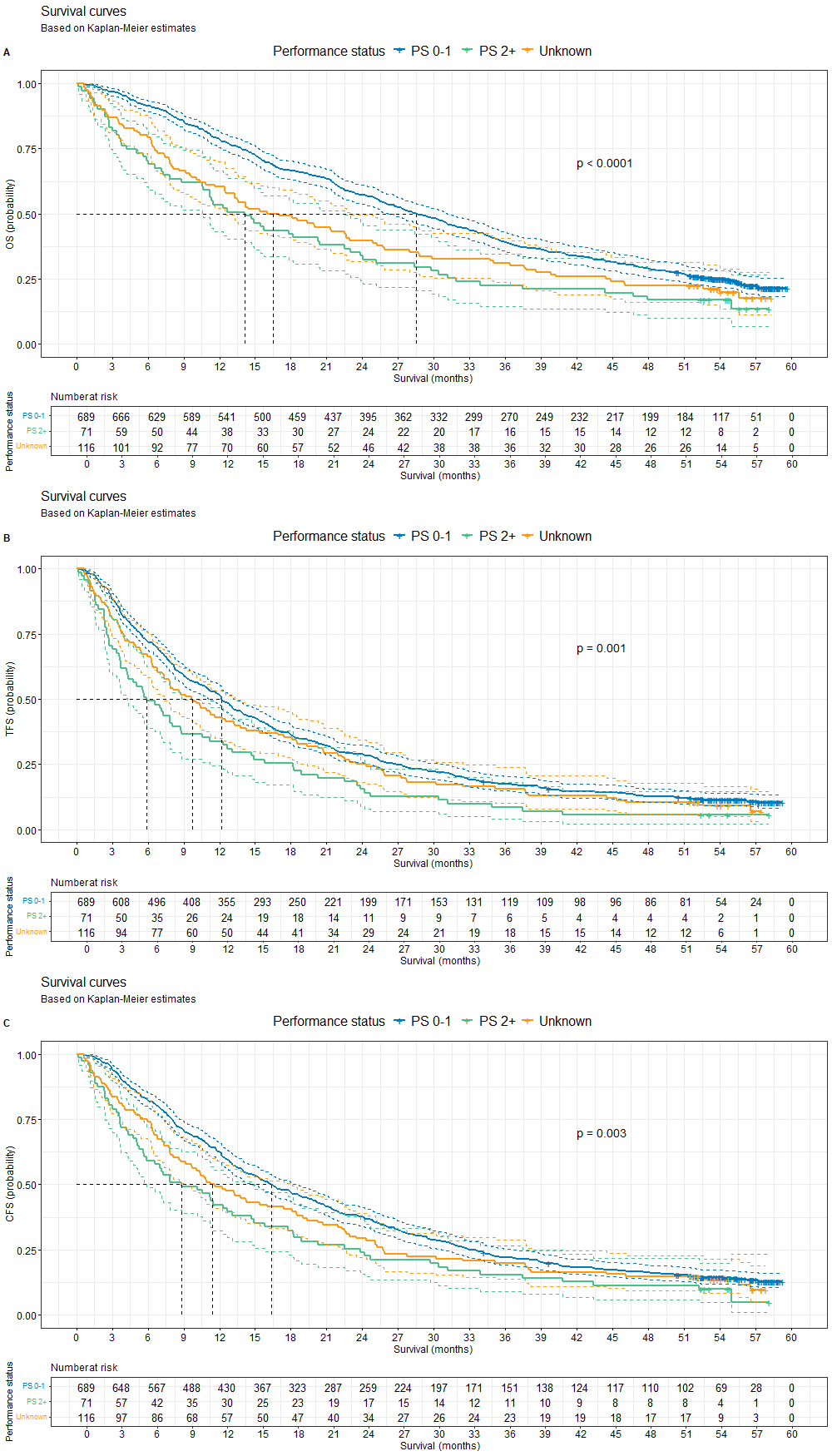


Table S1 – Post-discontinuation therapy amongst the real-world cohort that received abemaciclib plus fulvestrant for treatment of advanced HR+/HER2- breast cancer

| **Treatment Group** | **Regimen^[[1]](#footnote-1)^** | **Patient count (%)** |
| --- | --- | --- |
| Chemotherapy | Capecitabine | 201 (38.5%) |
| Chemotherapy | Paclitaxel | 64 (12.3%) |
| Chemotherapy | Eribulin | 23 (4.4%) |
| Chemotherapy | Vinorelbine | 15 (2.9%) |
| Chemotherapy | Carboplatin + Gemcitabine | 5 (1%) |
| Chemotherapy | Cyclophosphamide + Epirubicin | 5 (1%) |
| Chemotherapy | Carboplatin | 4 (0.8%) |
| Chemotherapy | Epirubicin | 4 (0.8%) |
| Chemotherapy | Carboplatin + Paclitaxel | 2 (0.4%) |
| Chemotherapy | Docetaxel | 2 (0.4%) |
| Chemotherapy | Gemcitabine + Paclitaxel | 2 (0.4%) |
| Chemotherapy | Capecitabine + Mitomycin | 1 (0.2%) |
| Chemotherapy | Cyclophosphamide + Epirubicin + Fluorouracil | 1 (0.2%) |
| Chemotherapy | Destiny Trial | 1 (0.2%) |
| Chemotherapy | Doxorubicin | 1 (0.2%) |
| Chemotherapy | Gemcitabine | 1 (0.2%) |
| Chemotherapy | Methotrexate + Mitomycin + Mitoxantrone | 1 (0.2%) |
| Chemotherapy | Trial Unspecified | 1 (0.2%) |
| Targeted therapy + Hormone therapy | Fulvestrant + Palbociclib | 48 (9.2%) |
| Targeted therapy + Hormone therapy | Everolimus + Exemestane | 46 (8.8%) |
| Targeted therapy + Hormone therapy | Fulvestrant + Ribociclib | 27 (5.2%) |
| Hormone therapy | Fulvestrant | 54 (10.3%) |
| Hormone therapy | Goserelin | 2 (0.4%) |
| Hormone therapy | Anastrozole | 1 (0.2%) |
| Hormone therapy | Exemestane | 1 (0.2%) |
| Hormone therapy | Leuprorelin Acetate | 1 (0.2%) |
| Chemotherapy + Targeted therapy | Docetaxel + Pertuzumab + Trastuzumab | 2 (0.4%) |
| Chemotherapy + Targeted therapy | Paclitaxel + Trastuzumab | 1 (0.2%) |
| Chemotherapy + Targeted therapy | Trastuzumab Emtansine | 1 (0.2%) |
| Targeted therapy | Olaparib | 1 (0.2%) |
| Targeted therapy | Trastuzumab | 1 (0.2%) |
| Non-breast treatment | Azacitidine + Venetoclax | 1 (0.2%) |
| Non-breast treatment | Osimertinib | 1 (0.2%) |

Table S2: Overall survival, treatment-free survival, and chemotherapy-free survival at 6, 12, 18, 24, 36 and 48-month intervals therapy amongst the real-world cohort that received abemaciclib plus fulvestrant for treatment of advanced HR+/HER2- breast cancer (N = 876)

| **Time period** | **Overall survival (%)** | **Treatment-free survival (%)** | **Chemotherapy-free survival (%)** |
| --- | --- | --- | --- |
| 6 months | 88% [95% CI: 86%, 90%] | 69% [95% CI: 66%, 73%] | 79% [95% CI: 77%, 82%] |
| 12 months | 74% [95% CI: 71%, 77%] | 49% [95% CI: 46%, 52%] | 59% [95% CI: 56%, 62%] |
| 18 months | 62% [95% CI: 59%, 65%] | 35% [95% CI: 32%, 39%] | 45% [95% CI: 42%, 48%] |
| 24 months | 53% [95% CI: 50%, 56%] | 27% [95% CI: 24%, 30%] | 35% [95% CI: 32%, 39%] |
| 36 months | 37% [95% CI: 34%, 40%] | 16% [95% CI: 14%, 19%] | 21% [95% CI: 19%, 24%] |
| 48 months | 27% [95% CI: 24%, 30%] | 12% [95% CI: 10%, 14%] | 16% [95% CI: 13%, 18%] |

1. The ‘Trial Unspecified’ regimen was grouped as chemotherapy following interrogation of drug level data. [↑](#footnote-ref-1)
